# Tunable CHA/AEI Zeolite Intergrowths with A Priori Biselective Organic Structure‐Directing Agents: Controlling Enrichment and Implications for Selective Catalytic Reduction of NOx

**DOI:** 10.1002/anie.202201837

**Published:** 2022-05-19

**Authors:** Estefanía Bello‐Jurado, Daniel Schwalbe‐Koda, Mathias Nero, Cecilia Paris, Toni Uusimäki, Yuriy Román‐Leshkov, Avelino Corma, Tom Willhammar, Rafael Gómez‐Bombarelli, Manuel Moliner

**Affiliations:** ^1^ Instituto de Tecnología Química Universitat Politècnica de València—Consejo Superior de Investigaciones Científicas (UPV-CSIC) Avenida de los Naranjos s/n 46022 València Spain; ^2^ Department of Materials Science and Engineering Massachusetts Institute of Technology Cambridge MA 02139 USA; ^3^ Department of Materials and Environmental Chemistry Stockholm University Stockholm University 10691 Stockholm Sweden; ^4^ Department of Chemical Engineering Massachusetts Institute of Technology Cambridge MA 02139 USA

**Keywords:** CHA/AEI intergrowths, Biselective OSDAs, Cu-containing zeolites, SCR-NOx, Machine Learning

## Abstract

A novel ab initio methodology based on high‐throughput simulations has permitted designing unique biselective organic structure‐directing agents (OSDAs) that allow the efficient synthesis of CHA/AEI zeolite intergrowth materials with controlled phase compositions. Distinctive local crystallographic ordering of the CHA/AEI intergrowths was revealed at the nanoscale level using integrated differential phase contrast scanning transmission electron microscopy (iDPC STEM). These novel CHA/AEI materials have been tested for the selective catalytic reduction (SCR) of NOx, presenting an outstanding catalytic performance and hydrothermal stability, even surpassing the performance of the well‐established commercial CHA‐type catalyst. This methodology opens the possibility for synthetizing new zeolite intergrowths with more complex structures and unique catalytic properties.

## Graphical abstract

Controlled CHA/AEI zeolite intergrowth materials were prepared with ab‐initio‐selected biselective organic structure‐directing agents (OSDAs), which were gleaned from high‐throughput simulation analysis of OSDA‐zeolite interactions. Efficient selective catalytic reduction of NOx was performed with the resulting materials. This method guides the preparation of zeolite intergrowths toward more complex structures with unique catalytic properties.

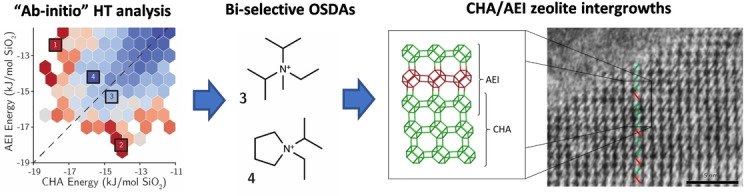

Cu‐containing zeolites with the CHA and AEI topologies are the two primary catalysts used for the SCR of NOx in automotive applications.[^1^, ^2^, ^3^, ^4^] On the one hand, high‐silica Cu‐AEI catalysts show higher hydrothermal stability compared to Cu‐containing CHA‐type materials for SCR applications.[^5^, ^6^] On the other hand, the preparation of Cu‐CHA catalysts have broader synthetic windows and use of less expensive OSDAs.[^7^, ^8^, ^9^, ^10^] Accordingly, the synthesis of high‐silica CHA/AEI intergrown zeolites could yield both preparative and operational advantages compared to their single‐phase counterparts. Current synthesis methods for high‐silica CHA/AEI intergrowths rely on the use of expensive OSDA mixtures, such as *N*,*N*,*N*‐trimethyladamantylammonium (TMAda) and *N*,*N*‐diethyl‐2,6‐dimethylpiperidinium (DEDMP),^[11]^ two well‐known OSDAs to synthesize pure CHA and AEI phases, respectively.[^12^, ^13^, ^14^] This dual‐OSDA approach was recently described for guiding the synthesis of high‐silica CHA/AFX intergrowths using 1,1′‐(1,4‐butanediyl)bis(1‐azonia‐4‐azabicyclo[2,2,2]octane) dication and TMAda as the specific OSDAs for AFX and CHA, respectively.^[15]^ Although the dual‐OSDA methodology can be extended to other industrially attractive zeolite intergrowths, two very specific OSDA molecules are needed under strictly fine‐tuned conditions to avoid the crystallization of the independent zeolite phases, increasing the preparation complexity and costs of the final materials. This problem is further exacerbated when trying to control the phase‐enrichment within the intergrown zeolite crystals, while avoiding phase segregation.

To circumvent these limitations, it is highly desirable to find a single OSDA that can direct the crystallization of the desired intergrowth composition. A standout example is the use of tetrabutylammonium (TBA) as a biselective OSDA to crystallize high‐silica MFI/MEL intergrowths.[^16^, ^17^, ^18^] MFI and MEL are very closely related structures formed exclusively by pentasil units and interconnected medium pores, requiring similar alkylammonium‐based cations.[^16^, ^18^] Unfortunately, the biselectivity of TBA to generate MFI/MEL intergrowths cannot be easily extended to more complex zeolite intergrowths exhibiting different pore topologies. Consequently, this leads to intensive and expensive experimental programs that rely on trial‐and‐error synthesis methods.^[19]^

Recently, we developed an “a priori” methodology based on high‐throughput simulations to quantify phase competition in zeolites.[^20^, ^21^] This toolkit was efficient not only for guiding the synthesis of pure zeolite frameworks by controlling OSDA sizes and shapes, but also for proposing single OSDA molecules with dual selectivity that facilitate zeolite intergrowth formation.^[22]^ In the latter case, the criteria for selecting OSDA candidates are: 1) similarly strong binding energies towards the frameworks forming the desired intergrowth, and weak binding energies towards undesired competing phases, and 2) favorable molecular shape that facilitates the crystallization of both frameworks. In addition, it is desirable to design OSDAs with low synthetic complexity to reduce synthesis costs of zeolites. This complexity can be quantified using cheminformatics or through expert inspection.^[20]^

To obtain detailed information of an intergrown material, it is mandatory to use characterization techniques that can adequately probe local intracrystalline structural features. Although transmission electron microscopy (TEM) can interrogate the local structure of zeolites, these materials are sensitive to the electron beam dose, requiring strict management of electron beam illumination to avoid damage. One approach to obtain atomic resolution images with an improved signal‐to‐noise ratios, is by using iDPC STEM. This method has recently been used to study the surface structure of MOFs^[23]^ and species inside zeolite pores.[^24^, ^25^, ^26^] A second emerging method is scanning electron diffraction (SED, also called 4DSTEM). In this approach, a small (<5 nm) electron probe with a small convergence angle is raster‐scanned across a sample and a diffraction pattern is acquired from each beam position to build up a map.^[27]^ The obtained data then reveal variations of the local crystallographic ordering at the nanoscale. SED has been used to elucidate the local structural ordering in MOFs,^[28]^ organic molecular crystals,^[29]^ polymers^[30]^ and biopolymers.^[31]^

Here we demonstrate the control of the CHA/AEI intergrowth enrichment when single, biselective OSDAs, as *N*‐ethyl‐*N*‐isopropyl‐*N*‐methylpropan‐2‐ammonium (OSDA3, Figure 1a) and 1‐ethyl‐1‐isopropylpyrrolidin‐1‐ium (OSDA4, Figure 1a), are selected from “a priori” high‐throughput simulations. The use of OSDA3 allows synthesizing CHA/AEI intergrowths with almost identical disordered low‐domains of CHA and AEI, whereas OSDA4 particularly promotes the presence of AEI‐enriched domains, as observed by iDPC STEM. These materials were Cu‐exchanged to evaluate their catalytic behavior for the NH_3_‐SCR of NOx, revealing that CHA/AEI intergrowths show enhanced catalytic activity and hydrothermal stability compared to well‐established Cu‐containing CHA catalyst.

**Figure 1:**
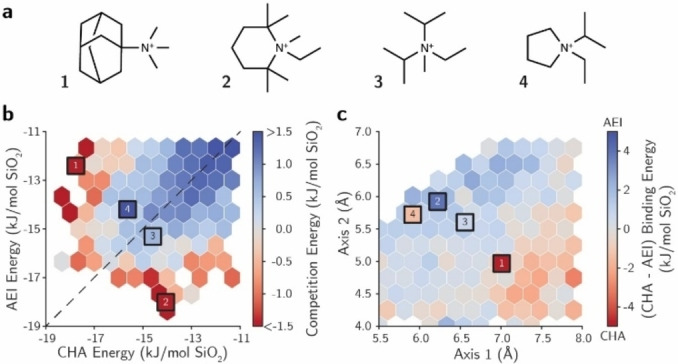
[a] OSDA candidates for CHA and AEI. [b] Comparison between binding energies of OSDAs in CHA and AEI. The colour of hexagons indicates the mean competition energy for all OSDAs within that area. [c] Relationship between OSDAs shapes and their binding energies toward CHA or AEI. Red (blue) colour indicates a binding energy more favourable towards CHA (AEI). Squares represent the energies/shapes of OSDAs shown in [a].

The best OSDAs to synthesize the pure crystalline CHA and AEI phases are TMAda (OSDA1 in Figure 1a) and *N*‐ethyl‐*N*‐methyl‐2,2,6,6‐tetramethylpiperidinium (OSDA2 in Figure 1a), respectively. These OSDAs show the best binding energies for each of the pure CHA and AEI phases (1 and 2 in Figure 1b) as calculated with molecular simulations.[^21^, ^32^] After filtering OSDAs according to the biselectivity criteria above and using the web‐based OSDB database,^[20]^ OSDA3 and OSDA4 are proposed as candidates for synthesizing CHA/AEI intergrowths. These templates exhibit binding energies for CHA and AEI that are close to the best ones, and also almost equal towards the two frameworks, as evidenced by their closeness to the equality line between CHA and AEI stabilization energies (see 3 and 4 in Figure 1b). This fact suggests that these OSDAs are excellent candidates to crystallize the desired CHA/AEI intergrowths due to their inferred selectivity toward either of these frameworks and ease of preparation (see OSDA synthesis procedures in the SI). Although the competition energies between the zeolites indicate a preferential crystallization of AEI with OSDA3 and of CHA with OSDA4 (Figures 1b and S2), the shape of the molecules is fundamental to control the zeolite selectivity,^[20]^ particularly within narrow ranges of favorable binding metrics such as those displayed by OSDA3 and OSDA4. With that in mind, the shape of the different OSDAs was quantified by projecting the three‐dimensional molecular conformer into a two‐dimensional plane, followed by measuring its major axes in the projection (SI for details). Then, the binding energies of the OSDAs are averaged over the shape domain, giving rise to Figure 1c.^[20]^ OSDA3 is aligned between OSDA1 and OSDA2, molecules that preferably stabilizes *cha* and *aei* cages, respectively. The shape of OSDA3 also lies in a region of biselectivity towards CHA/AEI, as demonstrated by the transition between CHA (red) and AEI (blue) selectivity domains in the shape space in Figure 1c.^[22]^ Thus, both binding energy and geometrical shape descriptors indicate that OSDA3 is a good candidate to obtain balanced CHA/AEI intergrowths. In contrast, the shape of OSDA4 lies in the domain of selectivity towards AEI, represented by the darker blue region in Figure 1c, and shows a geometrical shape similar to OSDA2, which is the molecule presenting the best host–guest fitting among the described organic molecules for the *aei* cage. Therefore, the geometrical descriptor suggests that OSDA4 is more favorable towards the *aei* cage compared to OSDA3 (Figure 1c), despite its slightly better energy towards CHA (Figure 1b). Based on the results from the “a priori” OSDA analysis, we surmise both OSDA3 and OSDA4 should exhibit excellent biselectivity binding metrics to guide the crystallization of the CHA/AEI intergrowth, with OSDA4 being more prone to direct the *aei* cage, thereby leading to higher AEI‐enrichments within the CHA/AEI intergrowths.

Considering these results, the synthesis of the CHA/AEI zeolite intergrowth was attempted using OSDA3 and OSDA4. The selected synthesis conditions were 1 SiO_2_ : 0.036 Al_2_O_3_ : 0.3 OSDA(OH) : 0.2 NaOH : 15 H_2_O, and 135–140 °C for crystallization (see synthesis details for CHA/AEI(3) and CHA/AEI(4) in the SI). The powder X‐ray diffraction (PXRD) patterns of the prepared materials are considerably different (Figure 2). The PXRD pattern of CHA/AEI(4) exhibits pronounced peaks between 16–19° and 21–22° 2θ angles, which are much weaker in the CHA/AEI(3) sample (Figure 2). When comparing the PXRD patterns of these as‐synthesized materials with those for the pure CHA and AEI materials (see synthesis descriptions in SI), it is seen that these peaks are absent in the CHA PXRD patterns (CHA in Figure 2), but present in that for AEI. This preliminary characterization indicates the presence of both CHA and AEI phases in the solids, but with noticeable differences in phase enrichment between them.

**Figure 2:**
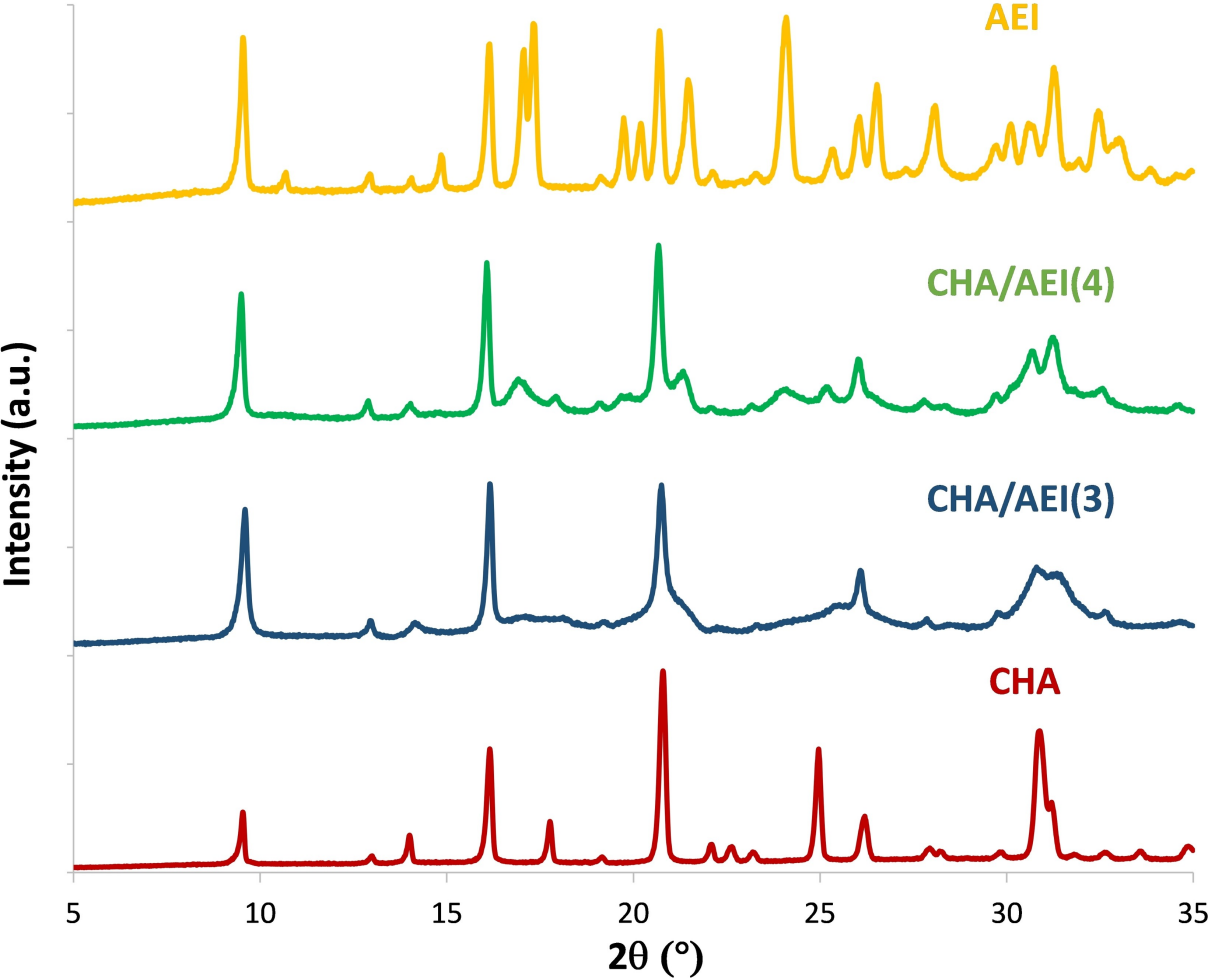
PXRD patterns of the as‐prepared CHA, AEI and CHA/AEI intergrowths.

To rule out the potential formation of a physical mixture of CHA and AEI crystals, both materials were first evaluated by Field Emission Scanning Electron Microscopy (FESEM) to visualize their particle morphologies. The solids present a uniform distribution of ∼300 nm crystals with analogous cubical morphologies, suggesting that the physical mixture of independent CHA and AEI crystals is not expected (CHA/AEI(3) and CHA/AEI(4) in Figure S3).

A detailed analysis of the crystals was carried out by iDPC STEM studies. The CHA/AEI(3) sample contains a disordered intergrowth between CHA and AEI (Figures 3a and 3b), with no evidence of any significant amounts of ordered AEI or CHA domains (Figures 3a and S4). In contrast, the CHA/AEI(4) sample contains large disordered domains (CHA/AEI intergrowth) and small domains of AEI structure (Figure 4a and S5). In almost every crystal, a twinning can be observed where the direction of the disorder is changing (circle, Figure 4b). The twinning is also confirmed by SED data that allow for the analysis of electron diffraction data from different areas during post processing. Although diffraction from larger regions exhibit disorder in two directions (blue), subsets of the data show that the direction of the disorder changes at different parts of the particle (green) (Figure S6). Diffraction from areas as small as ∼7×7 nm in size (1 and 2 in Figure 4c) reveals that some areas show diffuse scattering, indicating disordered domains (Figure 4c) while others are ordered AEI structures with sharp reflections. These structures are distributed in different parts of the crystal, mostly disordered, but with small domains of AEI as can be visualized using virtual purple and yellow dark‐field images in Figure 4c. Twinning is rather common in this sample, in line with the existence of few small point defects at the interface (Figure 4b).

**Figure 3:**
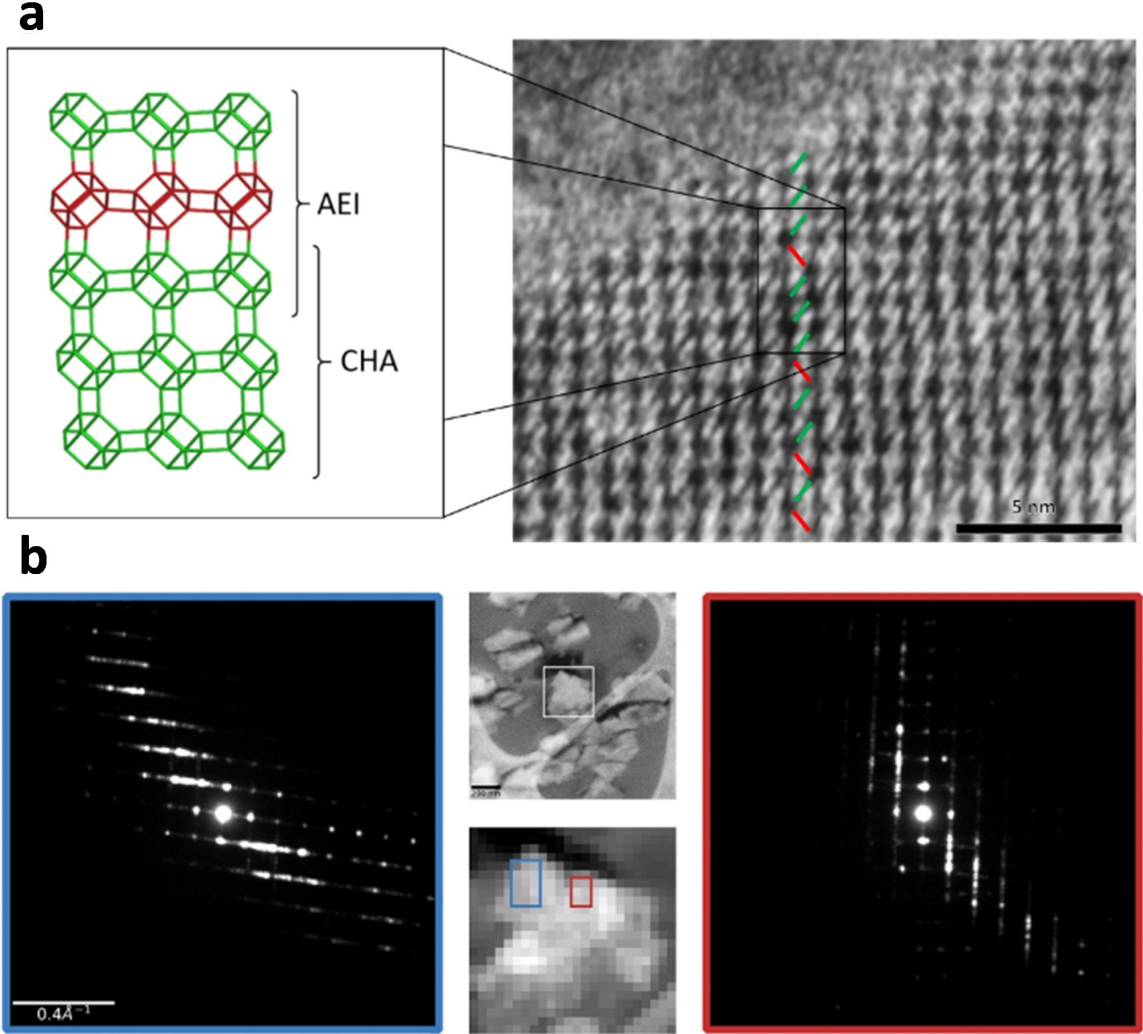
[a] iDPC‐STEM image of CHA/AEI(3). The image reveals the packing of the *d6r* units (green and red in [a]). Alternating ordering is consistent with AEI and domains of either red or green correspond to CHA. The images show intergrown structures where no domains contain more than 5 layers of ordered packing is found. [b] SED data aligned close to the [110] AEI direction, show diffuse streaking significative of layered intergrowth. Two different domains show a difference in the direction of the intergrowth related by a rotation of ∼94°.

**Figure 4:**
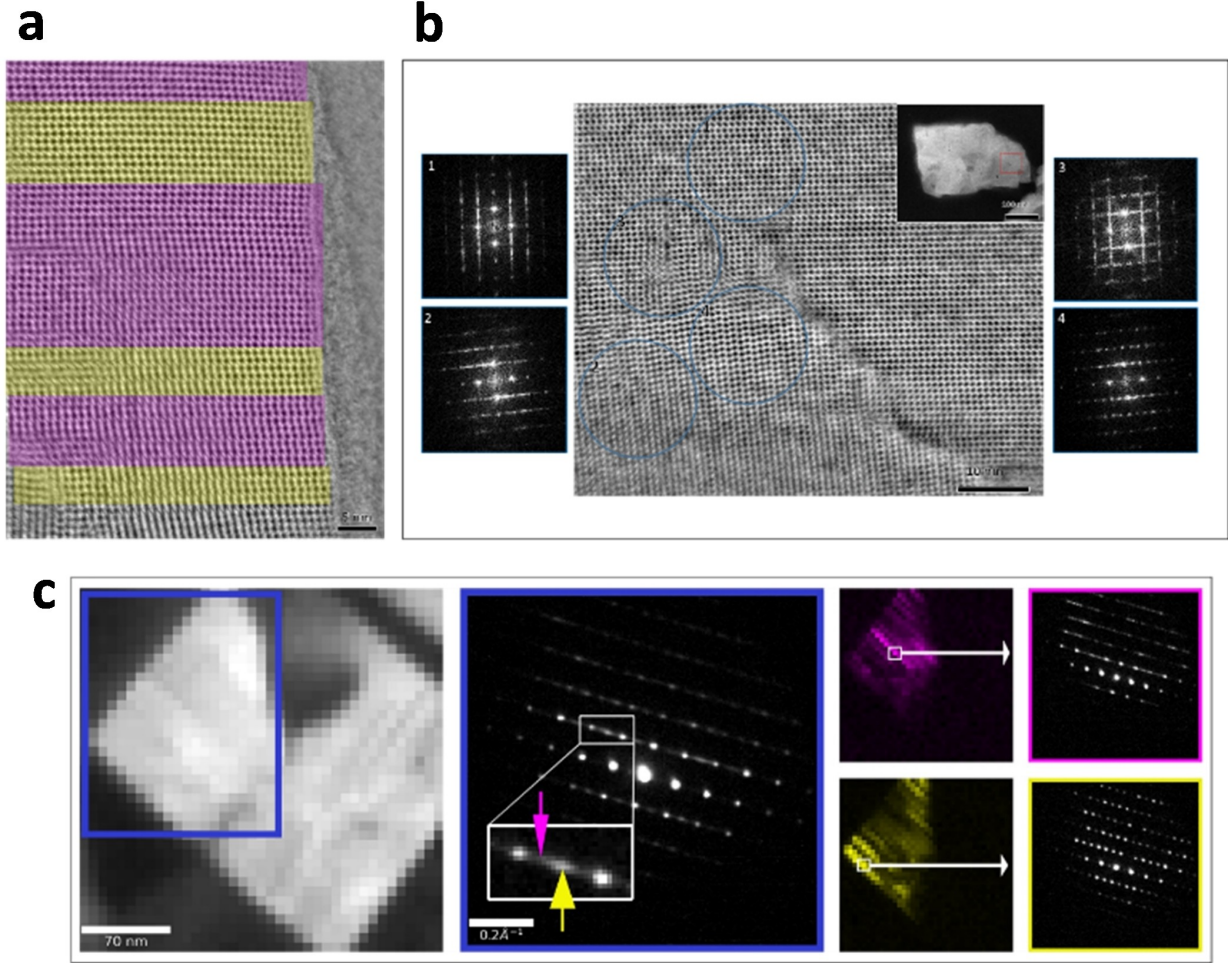
iDPC‐STEM images and SED data from the CHA/AEI(4) sample. [a] iDPC STEM image shows large domains of intergrowth (purple) together with domains of pure AEI structure (yellow). [b] iDPC STEM image shows twinning as indicated by the four Fourier transforms (FT) (1–3). The FTs shows that the material contains disordered domains rotated by ∼94° (1–3) as well as domains of ordered AEI structure (4). [c] SED data from a CHA/AEI(4) crystal aligned close to the [110] AEI direction, provides localized structural information. A map (left) where a diffraction pattern is acquired from each pixel. Averaged diffraction pattern (middle) extracted from the larger blue rectangle shows diffuse streaking confirming the presence of intergrowth in the material. Virtual dark field images (right) generated from two single pixels along the diffuse streaking (purple and yellow arrows) in the averaged diffraction pattern show that the crystal is heterogeneous. (right) Some parts are dominated by AEI type stacking (yellow) while other parts are disordered (purple). Two representative examples of diffraction patterns from single pixels of different characters are shown to the right.

Thus, the samples studied by iDPC STEM show a CHA/AEI type intergrowth, but each with different character: samples prepared with OSDA4 shows some preference for ordered AEI domains, whereas samples prepared with OSDA3 are mostly disordered (CHA/AEI ∼50/50). These findings are consistent with the previous PXRD data and with the expected outcomes from the molecular simulations and shape analysis. The difference in the character of the diffuse scattering can also be observed from SED data (Figure S7).

To evaluate the potential of these intergrown CHA/AEI materials for the selective reduction of NOx with ammonia (NH_3_‐SCR), additional physico‐chemical characterization was carried out. Chemical and FESEM analyses of the samples reveal similar chemical compositions (Si/Al∼9–10, Table 1) and particle sizes (∼300–400 nm, Figure S3). N_2_ adsorption experiments also indicate similar microporosity for all samples (∼0.28–0.29 cm^3^/g, Table 1), while the ^27^Al MAS NMR spectra of the samples reveal that most of the Al species remain in tetrahedral coordination after being calcined (see the main band centered at ∼50 ppm, Figure S8). In good agreement, the acid form of the CHA/AEI materials present analogous Brønsted acidities measured by temperature‐programmed desorption (TPD) of ammonia, both with a maximum desorption band centered at 450 °C (see Figure S9). Thus, considering that CHA/AEI(3) and CHA/AEI(4) display similar physico‐chemical properties, but differences on AEI enrichment, these intergrowth materials were selected for their catalytic evaluation on the NH_3_‐SCR of NOx. Pure CHA and AEI materials with similar physico‐chemical properties to CHA/AEI(3) and CHA/AEI(4) (Table 1), were evaluated for comparison purposes. The calcined materials were two‐time post‐synthetically cation exchanged, first with an aqueous solution of ammonium chloride to remove Na^+^ and later with an aqueous solution of copper acetate to incorporate ∼3 %wt of cationic copper species (SI).

**Table 1:** Zeolite chemical compositions in their as‐prepared forms and after Cu‐exchange. The textural properties have been measured by N_2_ adsorption on the Na‐containing calcined materials (before ammonium and Cu exchange treatments).

	ICP a.p		ICP after Cu‐exc		Textural properties		
Sample	Si/Al	Na (%wt)	Cu (%wt)	Na (%wt)	BET (m^2^/g)	Microp. Area (m^2^/g)	Mic. Vol. (cm^3^/g)
CHA	8.7	1.7	2.7	0.0	599	598	0.29
AEI	9.4	1.6	3.1	0.0	580	579	0.28
CHA/AEI(3)	10.2	1.3	3.1	0.0	608	591	0.28
CHA/AEI(4)	8.8	2.0	3.2	0.1	598	594	0.28

The Cu‐zeolites were tested under demanding reaction conditions, employing a feed composition of 500 ppm of NO, 500 ppm of NH_3_, 5 % of water and 7 % of O_2_, a wide range of reaction temperatures (170–550 °C), and a gas hourly space velocity (GHSV) as high as 450,000 ml/(h.g_cat_). The catalytic performance of the fresh Cu‐containing materials synthesized in this work indicates excellent NO conversion values for the four materials at intermediate temperatures (250–450 °C, Figure 5a), and similar low levels of N_2_O selectivities (Figure 5d). Interestingly, the intergrowth CHA/AEI materials show a remarkable improved catalytic profile compared to CHA‐type catalyst when subjected to severe high‐temperature steam treatments (see Figures 5b‐5e and 5c–5 f), particularly at 850 °C for 13 h (Figure 5c‐5 f). These results demonstrate that the presence of the AEI phase along the CHA/AEI intergrown crystals introduces an improved catalyst stability compared to the pure CHA‐type catalysts, approaching the performance of the highly resistant AEI‐type catalysts (Figure 5c).[^5^, ^6^]

**Figure 5:**
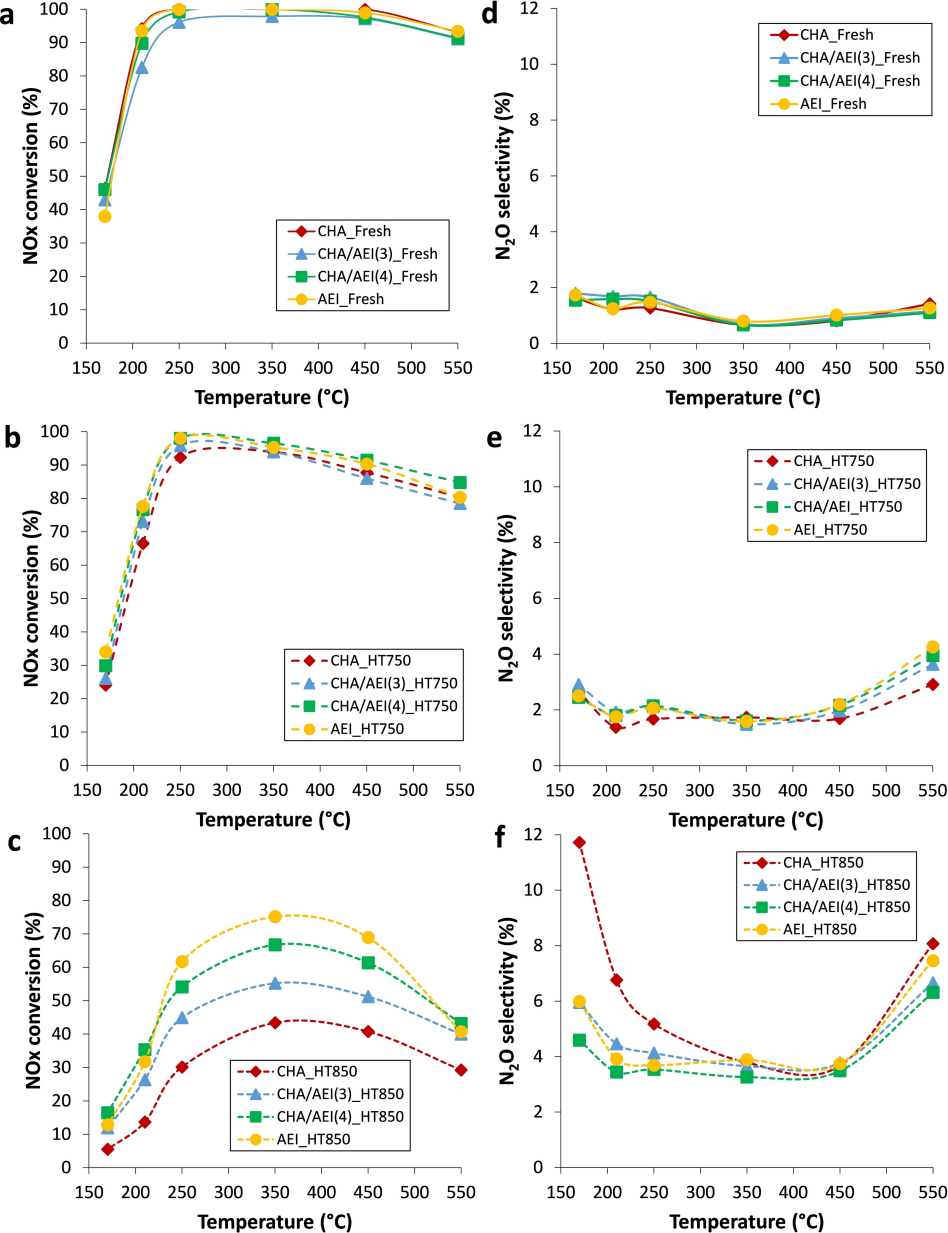
Catalytic results for the NH_3_‐SCR of NOx. [a–c] NO conversion values achieved with fresh [a] and aged catalysts at 750 °C [b] and 850 °C [c]. [d–f] N_2_O selectivities achieved with fresh [d] and aged catalysts at 750 °C [e] and 850 °C [f].

The “a priori” designed CHA/AEI intergrowth materials developed here open the possibility of rationalizing the preparation of highly resistant Cu‐zeolite catalysts, for instance with similar performance to the commercial AEI‐type catalysts, but employing simpler OSDAs than those traditionally required for the synthesis of the pure AEI phase. This fact can introduce substantial preparation and operational advantages, particularly when attempting to scale‐up the synthesis process of zeolite‐type catalysts. It is also worth noting that this methodology could be easily extended to the preparation of other zeolite intergrowths with high potential applicability in other industrial and environmental processes.^[22]^

## Conflict of interest

The authors declare no conflict of interest.

## Supporting information

As a service to our authors and readers, this journal provides supporting information supplied by the authors. Such materials are peer reviewed and may be re‐organized for online delivery, but are not copy‐edited or typeset. Technical support issues arising from supporting information (other than missing files) should be addressed to the authors.

Supporting Information

## Data Availability

The data that support the findings of this study are available in the supplementary material of this article.
