# Supplementary material for: Tunable CHA/AEI Zeolite Intergrowths with A Priori Biselective Organic Structure‐Directing Agents: Controlling Enrichment and Implications for Selective Catalytic Reduction of NOx
Source: Angew Chem Int Ed Engl. 2022 May 19;61(28):e202201837. doi: 10.1002/anie.202201837 (PMC9401568; doi:10.1002/anie.202201837)
Supplement: Supplementary file 1 — Supporting Information [file ANIE-61-0-s001.pdf]

Angewandte Chemie
Eine Zeitschrift der Gesellschaft Deutscher Chemiker

Supporting Information

Tunable CHA/AEI Zeolite Intergrowths with A Priori Biselective Organic Structure-Directing Agents: Controlling Enrichment and Implications for Selective Catalytic Reduction of NOx

E. Bello-Jurado, D. Schwalbe-Koda, M. Nero, C. Paris, T. Uusimäki, Y. Román-Leshkov, A. Corma, T. Willhammar, R. Gómez-Bombarelli*, M. Moliner**

1.- Theoretical section

The simulation data for all OSDAs was obtained from [1], and extended to OSDA4 using the same parameters. Specifically, force field calculations were performed using the General Utility Lattice Program (GULP) version 5.1.1 [2,3] using the GULPy package.[4] The Dreiding force field [5] was used to model dispersion interactions between pure-silica zeolites and OSDAs, which is able to recall past literature and experimental results,[1] in addition to correlating well with density functional theory calculations.[4]

Initial zeolite structures were obtained from the International Zeolite Association (IZA) database and optimized using the Sanders-Leslie-Catlow (SLC) force field.[6] Conformers for OSDAs were generated using RDKit [7] with the MMFF94 force field.[8,9]

OSDA-zeolite poses were produced using the Voronoi docking algorithm in the VOID package.[10] Binding energies between zeolites and OSDAs were computed using the frozen pose method.[4]

The shape of an OSDA was described by projecting the nuclear coordinates into a 2D space based on a principal component analysis (PCA).[1] The range of the distribution of points projected in each principal component is denoted as one of the principal axes of the conformer, with Axis 1 being the larger component. This shape descriptor correlates with synthetic accessibility of zeolites,[1] and has been proven useful for the design of biselective intergrowths beyond CHA/AEI.[11]

2.- Experimental Section

2.1.- Synthesis of the organic structure-directing agents (OSDA)

2.1.1.- *N,N,N*-trimethyl-1-adamantylammonium hydroxide (TMAda)

The aqueous solution of *N,N,N*-trimethyl-1-adamantylammonium hydroxide (25%wt) has been purchased from Sachem.

2.1.2.- Synthesis of *N,N*-diethyl-2,6-dimethylpiperidinium hydroxide (DEDMP)

Step 1: *(cis)*-2,6-dimethylpiperidine (0.84 mol, 95.12 g) was dissolved in acetone (400 ml). Then, the solution was cold at 0°C with an ice-bath. Under stirring, a slowly addition (dropwise) of an excess of iodoethane (1.68 mol, 262.14 g) was made over 1 h. After the solution was stabilized at room temperature, the mixture was slowly heated up to 50 °C and left to react for 72 h. The organic salt (*N*-ethyl-2,6-dimethylpiperidinium iodide), formed as a white crystalline solid, was isolated from the reaction mixture by filtration under reduced pressure. Finally, the product was recrystallized using an acetone-ethyl acetate solution, filtered and dried by heating under reduced pressure.

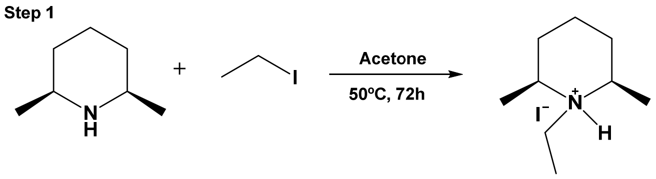

Step 2: *N*-ethyl-2,6-dimethylpiperidinium iodide (0.69 mol, 185.40 g) was dissolved at room temperature in 450 ml of distilled water. Under stirring, sodium hydroxide (0.69 mol, 27.6 g) was added, and the *N*-ethyl-2,6-dimethylpiperidine was formed in the upper part of the solution as an oily pale-yellow liquid. Finally, the amine was decanted, dried with anhydrous $MgSO_4$, and isolated by filtration.

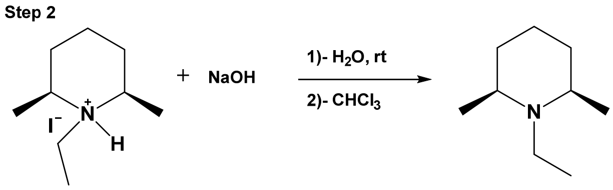

Step 3: *N*-ethyl-2,6-dimethylpiperidine (0.38 mol, 53.61 g) was dissolved in methanol (200 ml). The solution was cold at 0°C and then, under stirring, an excess of iodoethane was added (0.76 mol, 118, 54 g). Once the solution was stable at room temperature, it is slowly heated at reflux and left to react for 72 h. Finally, the solvent was removed by evaporation, and the product crystallized by addition of hot acetone.

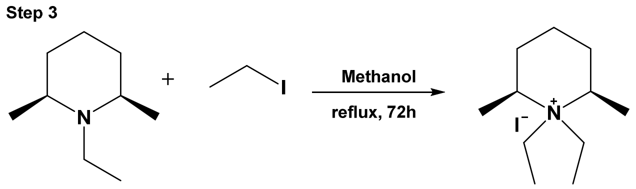

Step 4: 50 mmol of the iodide form of the template was dissolved in 110 ml of water. Then, 100 g of anion-exchange resin (Amberlite IRN-78) was added to the solution and kept under stirring for 24 h. Finally, the solution was collected by filtration and the obtained hydroxide form of the OSDA presented an exchange efficiency of at least 95%.

2.1.3.- Synthesis of *N*-ethyl-*N*-methyl-diisopropyl-ammonium hydroxide (OSDA3)

In a round-bottom flask, 278 mmol of *N*-ethyl-diisopropylamine (35.94 g) was dissolved in 150 ml of diethyl ether. The resulting solution was cold in an ice-bath and under continuous stirring, and 707 mmol of methyl iodide (100.32 g) was added dropwise in three aliquots over a 24 h period. Then, the solution was left to react one week at room temperature under stirring. When the reaction was completed, *N*-ethyl-*N*-methyl-diisopropyl-ammonium iodide precipitated as a white solid. The product was isolated by filtration and dried.

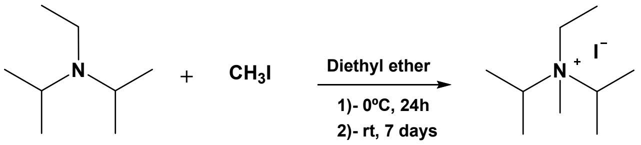

50 mmol of the iodide form of the template was dissolved in 110 ml of water. Then, 100 g of anion-exchange resin (Amberlite IRN-78) was added to the solution and kept under stirring for 24 hours. Finally, the solution was collected by filtration and the obtained hydroxide form of the OSDA presented an exchange efficiency of at least 95%.

2.1.4.- Synthesis of *1*-ethy-*1*-isopropylpyrrolidin-1-ium hydroxide (OSDA4)

A) Synthesis of 1-isopropylpyrrolidin-1-ium bromide

100 mmol of pyrrolidine (7.11 g) was dissolved in 100 ml of *2*-propanol and then 250 mmol (30.75 g) of *2*-bromopropane was added through small aliquots under stirring. The resulting solution was heated to 70°C and allowed to react for 24 h. Afterwards, the reaction mixture was cooled down to room temperature, and an ethyl acetate-acetone mixture was added to precipitate the organic salt. Finally, *1*-Isopropylpyrrolidin-*1*-ium bromide was isolated by filtration and recrystallized.

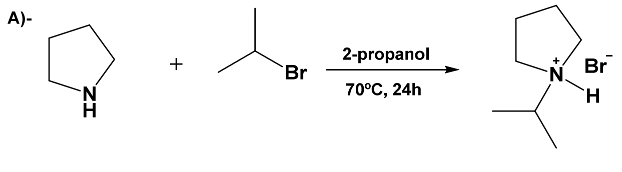

B) Synthesis of 1-isopropylpyrrolidine

74 mmol of *1*-isopropylpyrrolidin-*1*-ium bromide (14.38 g) was dissolved in 100 ml of distilled water, and 74 mmol of sodium hydroxide (2.96 g) was added under stirring. The resulting solution was kept one hour at room temperature. A biphasic mixture was obtained, in which *1*-isopropylpyrrolidine was present at the top phase. The compound was separated by decantation and, a liquid-liquid extraction was carried out with chloroform (2 x 50 ml) on the remaining aqueous phase to recover part of the dissolved amine. The two organic parts were combined, dried over anhydrous magnesium sulphate, filtered to remove the inorganic salt and, finally, the solvent was evaporated. *1*-Isopropylpyrrolidine was obtained as a thick yellow liquid.

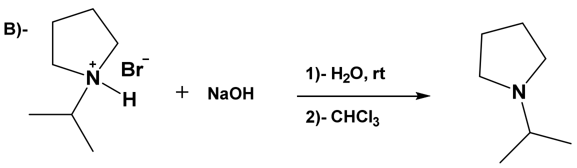

C) Synthesis of 1-ethyl-1-isopropylpyrrolidin-1-ium hydroxide

71 mmol of *1*-isopropylpyrrolidine (8.03 g) was dissolved in 60 ml of anhydrous methanol and, later, an excess of iodoethane (176 mmol, 27.5 g) was added dropwise under stirring at room temperature. The resulting mixture was slowly heated to 60°C and allowed to react for 72 h. Afterwards, the solvent was evaporated and *1*-ethyl-*1*-isopropylpyrrolidin-1-ium iodide was precipitated by adding ethyl ether, separated by filtration and dried.

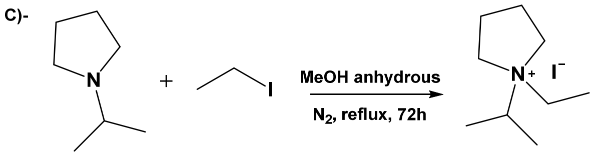

50 mmol of *1*-ethyl-*1*-isopropylpyrrolidin-*1*-ium iodide was dissolved in 110 ml of water. Then, 100 g of anion-exchange resin (Amberlite IRN-78) was added to the previous solution and kept stirring for 24 hours. Finally, the solution was collected by filtration and the hydroxide form of *1*-ethyl-*1*-isopropylpyrrolidin-1-ium presented an exchange efficiency of at least 95%.

2.2.- Synthesis of the zeolites

2.2.1.- Synthesis of CHA

3.8 g of FAU zeolite (FAU, CBV720 with Si/Al=14, Zeolyst, Lot number: 72004003128) was added to 10.1 g of a 25 wt% aqueous solution of TMAda(OH). The mixture was stirred for 10 minutes for homogenization. Afterwards, 3.6 g of a 20 wt% aqueous solution of sodium hydroxide was added, and the synthesis mixture was maintained under stirring the required time to evaporate the excess of water until achieving the desired gel concentration.

The final gel composition was: 1 SiO_2 : 0.036 Al_2O_3 : 0.2 TMAda(OH) : 0.3 NaOH : 15 H_2O.

The resultant gel was charged into a stainless steel autoclave with a Teflon liner. The crystallization was then conducted at 150°C for 7 days under dynamic conditions. The solid product was filtered, washed with abundant water, and dried at 100°C. The solid was calcined at 580°C for 5 h in air to remove the occluded organic molecules.

2.2.2.- Synthesis of AEI

1.8 g of FAU zeolite (FAU, CBV720 with Si/Al=14, Zeolyst, Lot number: 72004003128) was added to 14.9 g of a 9.45 wt% aqueous solution of DEDMP(OH). The mixture was stirred for 10 minutes for homogenization. Afterwards, 1.0 g of a 20 wt% aqueous solution of sodium hydroxide was added, and the synthesis mixture was maintained under stirring the required time to evaporate the excess of water until achieving the desired gel concentration.

The final gel composition was: 1 SiO_2 : 0.036 Al_2O_3 : 0.3 DEDMP(OH) : 0.2 NaOH : 15 H_2O.

The resultant gel was charged into a stainless steel autoclave with a Teflon liner. The crystallization was then conducted at 135°C for 7 days under dynamic conditions. The solid product was filtered, washed with abundant water, and dried at 100°C. The solid was calcined at 580°C for 5 h in air to remove the occluded organic molecules.

2.2.3.- Synthesis of CHA/AEI(3)

2.2 g of FAU zeolite (FAU, CBV720 with Si/Al=14, Zeolyst, Lot number: 72004003128) was added to 22.1 g of a 6.6 wt% aqueous solution of OSDA3(OH). The mixture was stirred for 10 minutes for homogenization. Afterwards, 1.2 g of a 20 wt% aqueous solution of sodium hydroxide was added, and the synthesis mixture was maintained under stirring the required time to evaporate the excess of water until achieving the desired gel concentration.

The final gel composition was: 1 SiO_2 : 0.036 Al_2O_3 : 0.3 OSDA3(OH) : 0.2 NaOH : 15 H_2O.

The resultant gel was charged into a stainless steel autoclave with a Teflon liner. The crystallization was then conducted at 140°C for 5 days under dynamic conditions. The solid product was filtered, washed with abundant water, and dried at 100°C. The solid was calcined at 580°C for 5 h in air to remove the occluded organic molecules.

2.2.4.- Synthesis of CHA/AEI(4)

0.9 g of FAU zeolite (FAU, CBV720 with Si/Al=14, Zeolyst, Lot number: 72004003128) was added to 9.2 g of a 6.5 wt% aqueous solution of OSDA4(OH). The mixture was stirred for 10 minutes for homogenization. Afterwards, 0.5 g of a 20 wt% aqueous solution of sodium hydroxide was added, and the synthesis mixture was maintained under stirring the required time to evaporate the excess of water until achieving the desired gel concentration.

The final gel composition was: 1 SiO_2 : 0.036 Al_2O_3 : 0.3 OSDA4(OH) : 0.2 NaOH : 15 H_2O.

The resultant gel was charged into a stainless steel autoclave with a Teflon liner. The crystallization was then conducted at 135°C for 6 days under dynamic conditions. The solid product was filtered, washed with abundant water, and dried at 100°C. The solid was calcined at 580°C for 5 h in air to remove the occluded organic molecules.

2.3.- Cu-exchange treatments

The calcined solids were first exchanged with a 2M aqueous solution of ammonium nitrate (NH_4Cl, Sigma-Aldrich, 99% by weight) with a liquid/solid ratio of 10, maintaining the mixture at 80°C for 2 hours under agitation. Afterwards, the solids were recovered by filtration. 0.3 g of the previous ammonium-exchanged zeolites was introduced in 30 ml of an aqueous solution of $Cu(CH_3COO)_2{\cdot}H_2O$ [28.3 mg of $Cu(CH_3COO)_2{\cdot}H_2O$ dissolved in 30 ml of water], maintaining a solid/liquid ratio of 10 g/l at 80°C for 24 h. Finally, the solids were filtered and washed with distilled water, dried and calcined at 550°C in air for 4 h.

2.4.- Hydrothermal ageing treatments of Cu-exchanged zeolites

The Cu-exchanged zeolites were subjected to a hydrothermal treatment at 750 or 850°C using a 300 ml/min flow rate with 10% water, 10% of O_2 and balanced with nitrogen for 13 hours.

2.5.- Characterization

Powder X-ray diffraction (PXRD) measurements were performed with a multi sample Philips X'Pert diffractometer equipped with a graphite monochromator, operating at 40 kV and 35 mA, and using Cu Kα radiation (λ = 0.1542 nm).

Chemical analyses were carried out in a Varian 715-ES ICP-Optical Emission spectrometer, after solid dissolution in HNO_3/HCl/HF aqueous solution.

^{27}Al MAS NMR spectra were recorded at room temperature with a Bruker AV 400 spectrometer at 104.2 MHz with a spinning rate of 10 kHz and 9° pulse length of 0.5 μs with a 1 s repetition time. ^{27}Al chemical shift was referred to $Al^{3+}(H_2O)_6$.

Nitrogen adsorption isotherms at -196°C were measured on a Micromeritics ASAP 2020 with a manometric adsorption analyser to determinate the textural properties of the samples.

The morphology of the samples was studied by field emission scanning electron microscopy (FESEM) using a ZEISS Ultra-55 microscope.

The sample was prepared for transmission electron microscopy (TEM) studies using ultra microtomy in order to obtain thin sections and to access the desired crystallographic direction for imaging. The zeolite powder was dried in oven overnight and embedded in an epoxy resin (Agar Low Viscosity Resin) which was hardened at 60°C for 24 h. Sectioning was performed using a Leica Ultracut UCT with a 45° diamond knife from Diatome to an estimated thickness of 50 nm. After cutting, the sections were transferred to holey carbon coated copper grids. Scanning transmission electron microscopy (STEM) images were obtained using a ThermoFisher ThemisZ double aberration-corrected TEM using an integrated differential phase contrast (iDPC) detector. The TEM was operated at an accelerating voltage of 300 kV. The image contrast was formed. A high-pass filter was applied to the iDPC images in order to reduce low frequency noise. The STEM images were acquired using an electron beam dose current of 20 pA, a convergence angle of 16 mrad and a dwell time of 10 μs. The sample was dried in vacuum at 180°C during 3 h prior to data acquisition in order to remove adsorbed water and enhance stability. The SED data were acquired using the in-house build software suite St4DeM, capable of acquiring 300 frames/s

using the Gatan Oneview camera with software synchronization. St4DeM is written within the Digital Micrograph SDK environment and is successfully tested with Thermo Fischer 300 kV Themis and JEOL 2100F microscopes.[12] The SED data was analyzed using Pyxem.[13]

NH_3-TPD experiments were carried out in a Micromeritics 2900 apparatus. A calcined sample (100 mg) was activated by heating to 400°C for 2 h in an oxygen flow and for 2 h in argon flow. Subsequently, the samples were cooled down to 176°C, and NH_3 was adsorbed. The NH_3 desorption was monitored with a quadrupole mass spectrometer (Balzers, Thermo Star GSD 300T) while the temperature of the sample was ramped at 10°C min^{-1} in helium flow.

2.6.- NH_3-SCR catalytic test

The catalytic activity was evaluated for the catalytic reduction of NOx with NH_3 in a fixed bed, quartz tubular reactor with 1.2 cm inner diameter. 40 mg sieve fractionated catalyst was used diluted in 1.2 g of silicon carbide. The catalysts were introduced in the reactor and heated up to 550°C in a 300 mL/min flow of nitrogen and maintained at this temperature for one hour. Afterwards, the feed was admitted over the catalyst while maintaining a flow of 300 mL/min. The feed composition for the catalytic tests performed over the Cu-containing catalysts was 500 ppm NOx, 550 ppm NH_3, 7% O_2 and 5% H_2O. The reaction temperature was decreased stepwise between 550 and 170°C. The conversion of NOx was measured under steady state conversion at each temperature using a chemiluminescence detector (Thermo 62C). The NH_3 concentration was monitored in a UV spectroscopy gas analyser from Tethys Instrument, Model EXM400. The N_2O concentration was measured with an infrared spectroscopy analyser from Servomex, Model 4900. The NOx conversion and the product selectivities were calculated by Equations (1)–(3):

$$NOx\ conversion\ (\%) = \frac{NOx_{in} - NOx_{out}}{NOx_{in}} \times 100 \qquad (1)$$

$$N_2O\ selectivity\ (\%) = \frac{N_2O_{out}}{NOx_{in} - NOx_{out}} \times 100 \qquad (2)$$

$$N_2\ selectivity\ (\%) = 100 - N_2O \times 100 \qquad (3)$$

where “in” corresponds to the inlet concentration, and “out” the outlet concentration.

Supplementary Figures

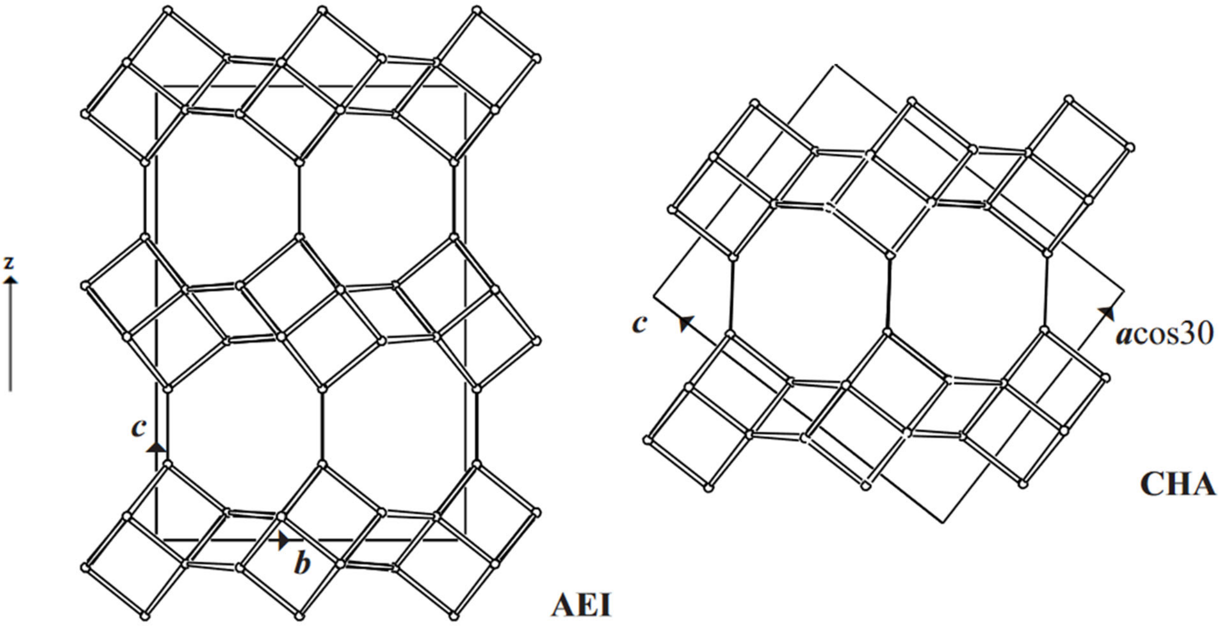

Figure S1. Structures of AEI (left) and CHA (right) zeolites.

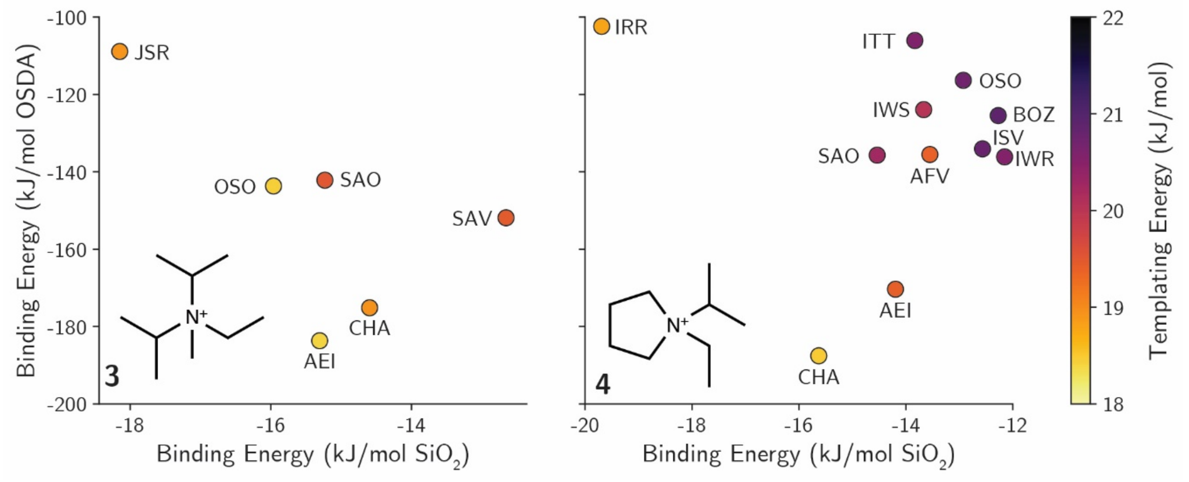

Figure S2. Phase competition for OSDA3 and OSDA4. The binding energy per OSDA (kJ/mol OSDA) and SiO_2 (kJ/mol SiO_2) are shown as independent parameters. The templating energy is given in kJ/mol.

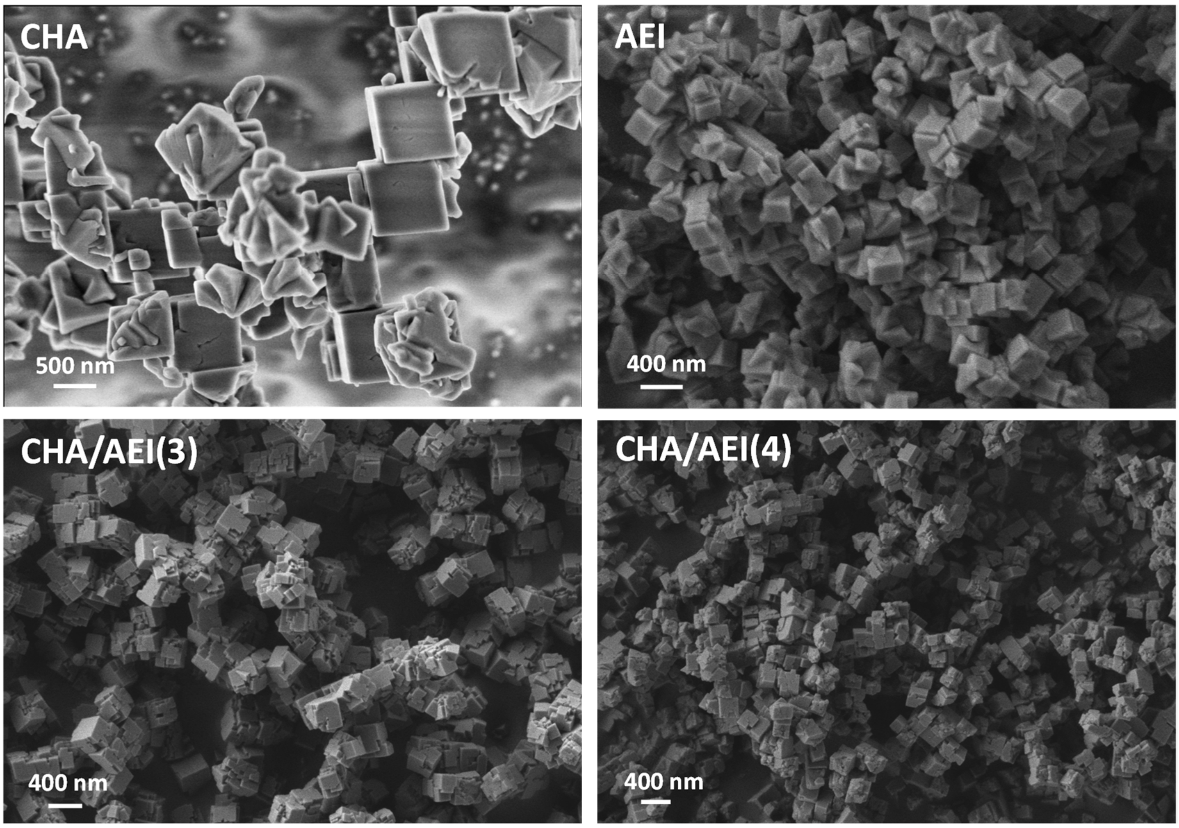

Figure S3. FE-SEM images of the different zeolites.

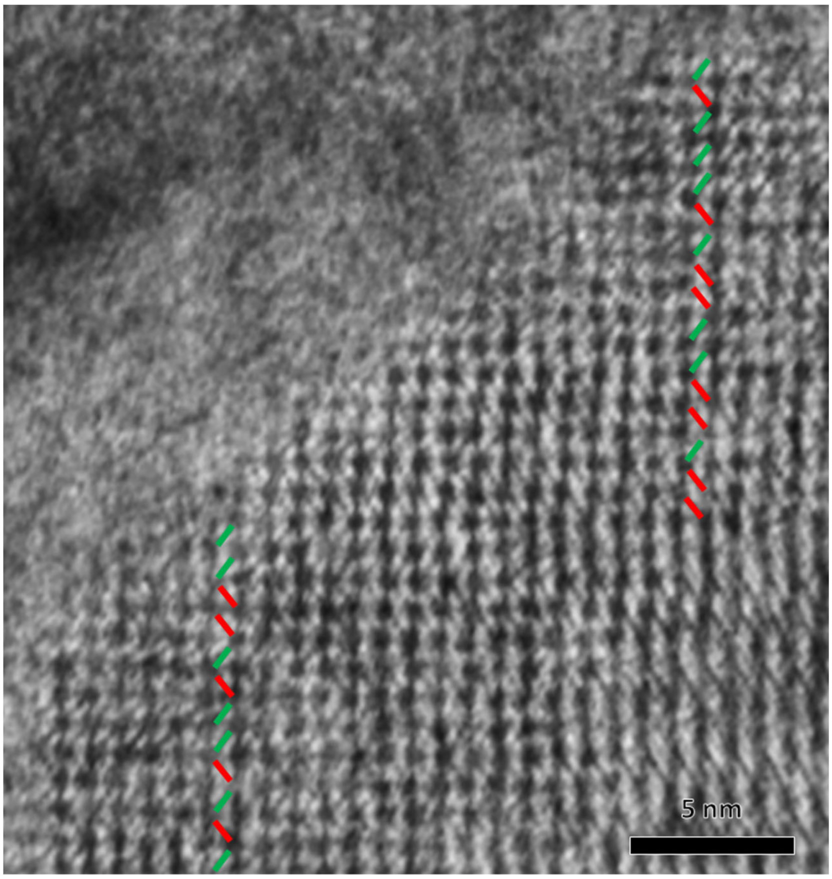

Figure S4. Additional iDPC STEM images from sample CHA/AEI(3) showing the disordered nature of the layer sequence.

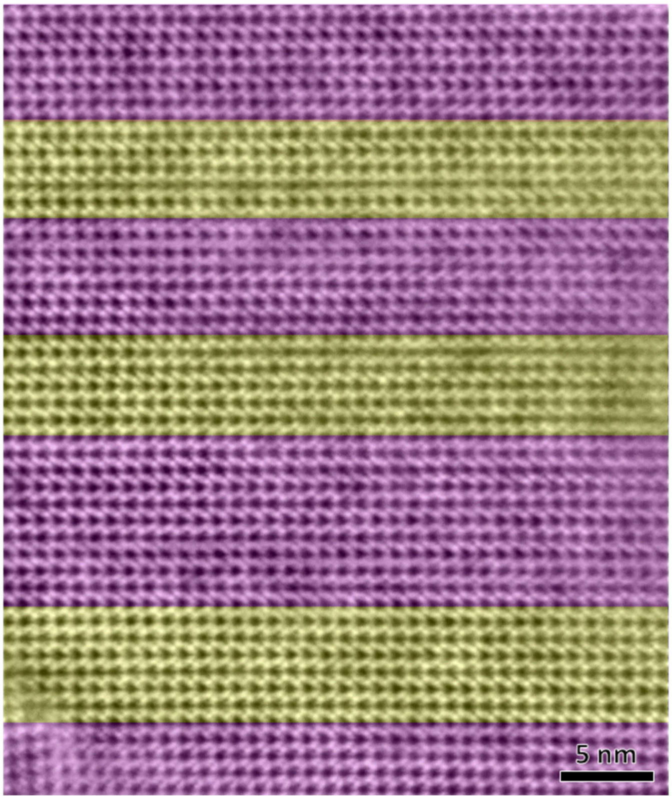

Figure S5. Additional iDPC-STEM image from sample CHA/AEI(4) revealing the presence of smaller domains of intergrowth (purple) together with domains of pure AEI structure (yellow).

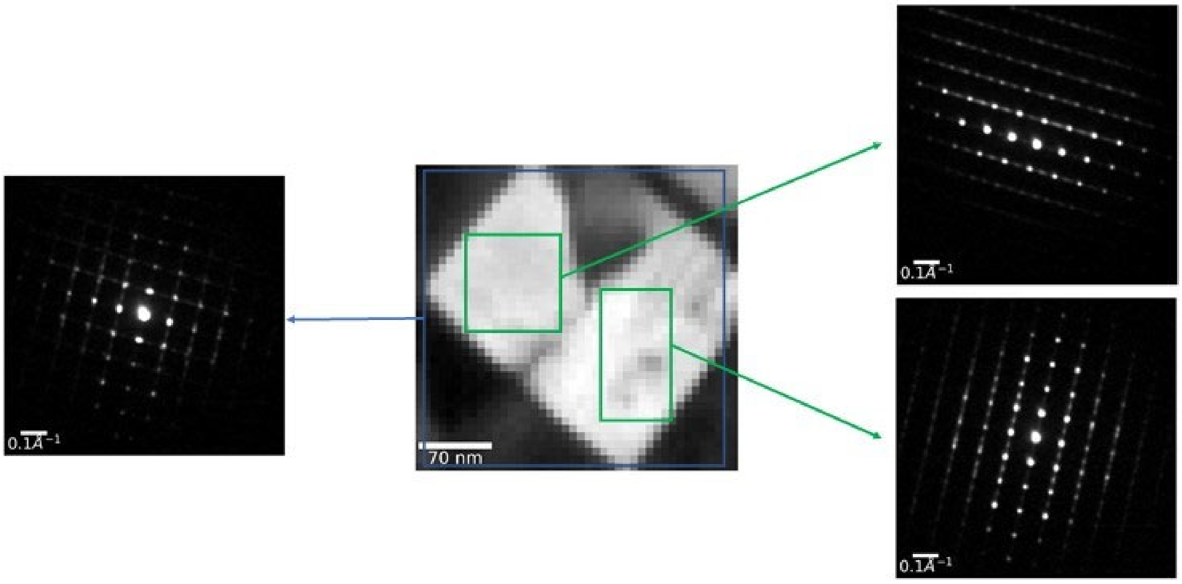

Figure S6. Scanning electron diffraction data from a CHA/AEI(4) crystal. Averaged data from the entire region (marked in dark blue) shows diffuse streaking in two directions related by an angle of 94º. Diffraction data extracted from each of the two sub domains (shown in green) shows that each of the domains has stacking disorder along one dimension.

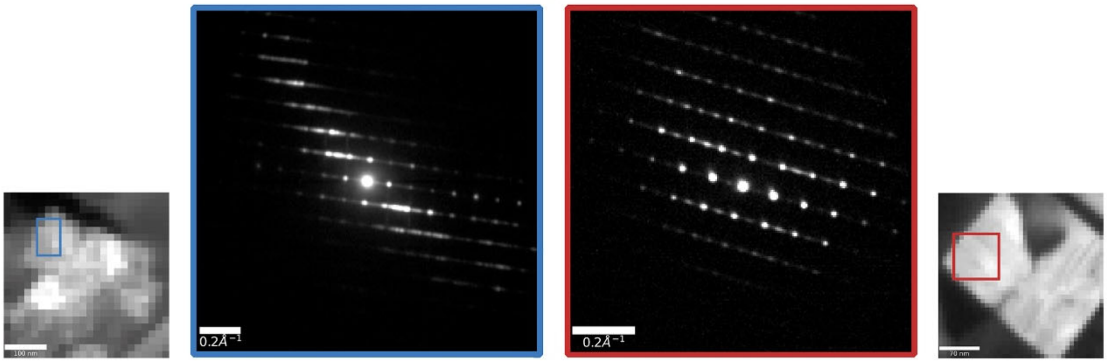

Figure S7. Comparison of scanning electron diffraction data from samples CHA/AEI(3) (blue, left) and CHA/AEI(4) (red, right). The maps on either side show the crystal morphology and the region from which the average diffraction patterns are formed is marked by a blue and red square respectively. The intensity along the diffuse streaks have different character. Sample CHA/AEI(4) shows enhanced intensity halfway between the sharp reflections indicating an enrichment in AEI-type stacking. Sample CHA/AEI(3) shows diffuse streaking of more continuous intensity.

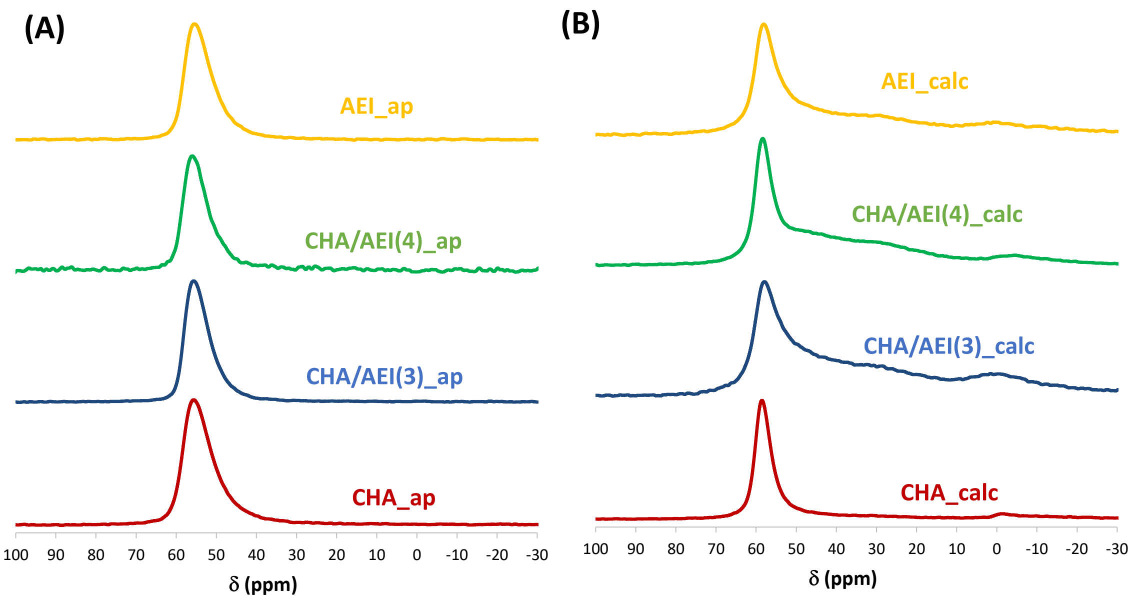

Figure S8. ^{27}Al MAS NMR spectra of the as-prepared (A) and calcined (B) CHA, AEI and CHA/AEI intergrowth zeolites

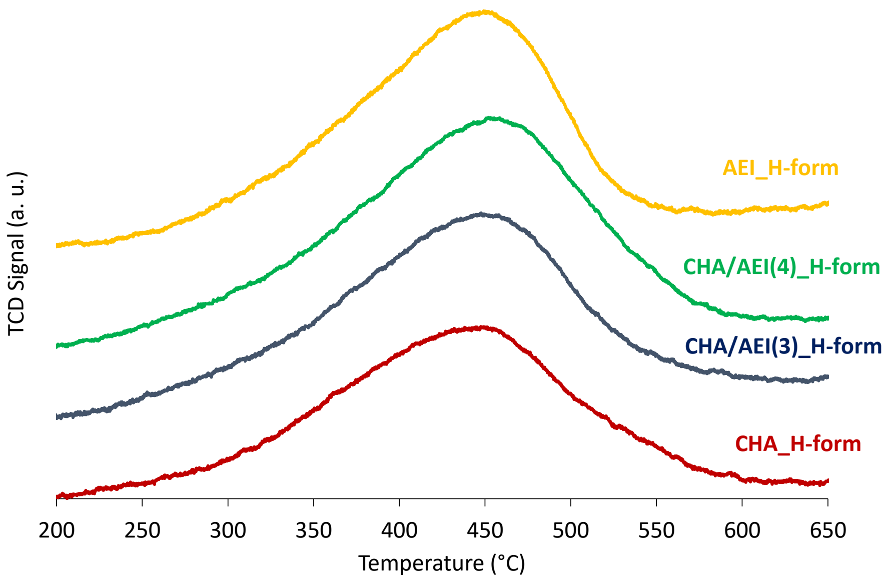

Figure S9. NH_3-TPD profiles of the different small-pore zeolites in their acid forms

References:

[1] D. Schwalbe-Koda, S. Kwon, C. Paris, E. Bello-Jurado, Z. Jensen, E. Olivetti, T. Willhammar, A. Corma, Y. Román-Leshkov, M. Moliner, R. Gómez-Bombarelli, *Science* **2021**, *374*, 308–315.

[2] J. D. Gale, *J. Chem. Soc. Faraday Trans.* **1997**, *93*, 629–637.

[3] J. D. Gale, A. L. Rohl, *Mol. Simul.* **2003**, *29*, 291–341.

[4] D. Schwalbe-Koda, R. Gómez-Bombarelli, *J. Chem. Phys.* **2021**, *154*, 174109.

[5] S. L. Mayo, B. D. Olafson, W. A. Goddard, *J. Phys. Chem.* **1990**, *94*, 8897–8909.

[6] M. J. Sanders, M. Leslie, C. R. A. Catlow, *J. Chem. Soc. Chem. Commun.* **1984**, 1271.

[7] G. Landrum, *Available www.rdkit.org (accessed Nov. 1, 2021)* **n.d.**

[8] T. A. Halgren, *J. Comput. Chem.* **1996**, *17*, 490–519.

[9] P. Tosco, N. Stiefl, G. Landrum, *J. Cheminform.* **2014**, *6*, 37.

[10] D. Schwalbe-Koda, R. Gómez-Bombarelli, *J. Phys. Chem. C* **2021**, *125*, 3009–3017.

[11] D. Schwalbe-Koda, A. Corma, Y. Román-Leshkov, M. Moliner, R. Gómez-Bombarelli, *J. Phys. Chem. Lett.* **2021**, *12*, 10689–10694.

[12] D. R. G. Mitchell, B. Schaffer, *Ultramicroscopy* **2005**, *103*, 319–332.

[13] D. N. Johnstone, P. Crout, J. Laulainen, S. Høgås, B. Martineau, T. Bergh, S. Smeets, S. Collins, J. Morzy, H. Ånes, E. Prestat, T. Doherty, T. Ostasevicius, M. Danaie, R. Tovey, *Zenodo* **2019**, DOI 10.5281/zenodo.3533653.
